# The Future of Ligand Engineering in Colloidal Semiconductor Nanocrystals

**DOI:** 10.1021/acs.accounts.0c00765

**Published:** 2021-02-26

**Authors:** Juliette Zito, Ivan Infante

**Affiliations:** † Department of Nanochemistry, Istituto Italiano di Tecnologia, Via Morego 30, 16163 Genova, Italy; ‡ Dipartimento di Chimica e Chimica Industriale, Università degli Studi di Genova, Via Dodecaneso 31, 16146 Genova, Italy

## Abstract

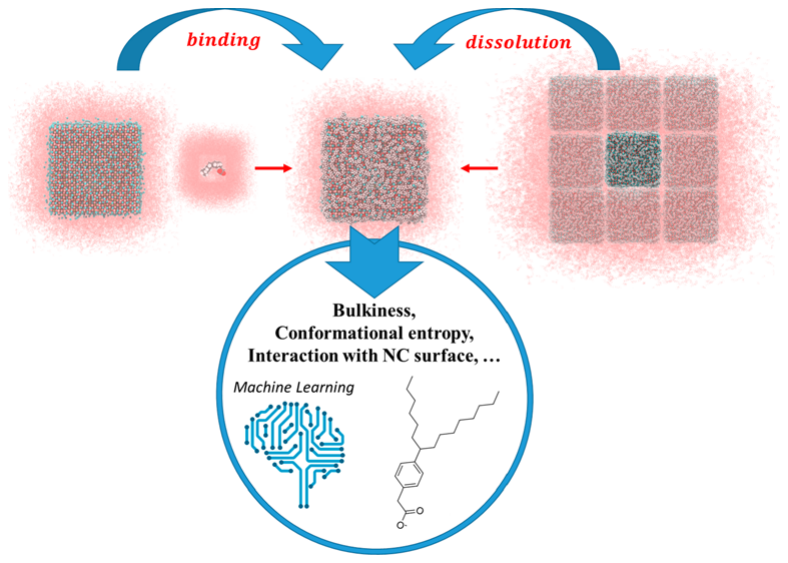

Next-generation colloidal semiconductor nanocrystals featuring enhanced optoelectronic properties and processability are expected to arise from complete mastering of the nanocrystals’ surface characteristics, attained by a rational engineering of the passivating ligands. This aspect is highly challenging, as it underlies a detailed understanding of the critical chemical processes that occur at the nanocrystal–ligand–solvent interface, a task that is prohibitive because of the limited number of nanocrystal syntheses that could be tried in the lab, where only a few dozen of the commercially available starting ligands can actually be explored. However, this challenging goal can be addressed nowadays by combining experiments with atomistic calculations and machine learning algorithms. In the last decades we indeed witnessed major advances in the development and application of computational software dedicated to the solution of the electronic structure problem as well as the expansion of tools to improve the sampling and analysis in classical molecular dynamics simulations. More recently, this progress has also embraced the integration of machine learning in computational chemistry and in the discovery of new drugs. We expect that soon this plethora of computational tools will have a formidable impact also in the field of colloidal semiconductor nanocrystals.

In this Account, we present some of the most recent developments in the atomistic description of colloidal nanocrystals. In particular, we show how our group has been developing a set of programs interfaced with available computational chemistry software packages that allow the thermodynamic controlling factors in the nanocrystal surface chemistry to be captured atomistically by including explicit solvent molecules, ligands, and nanocrystal sizes that match the experiments. At the same time, we are also setting up an infrastructure to automate the efficient execution of thousands of calculations that will enable the collection of sufficient data to be processed by machine learning.

To fully capture the power of these computational tools in the chemistry of colloidal nanocrystals, we decided to embed the thermodynamics behind the dissolution/precipitation of nanocrystal–ligand complexes in organic solvents and the crucial process of binding/detachment of ligands at the nanocrystal surface into a unique chemical framework. We show that formalizing this mechanism with a computational bird’s eye view helps in deducing the critical factors that govern the stabilization of colloidal dispersions of nanocrystals in an organic solvent as well as the definition of those key parameters that need to be calculated to manipulate surface ligands. This approach has the ultimate goal of engineering surface ligands in silico, anticipating and driving the experiments in the lab.

## Key References

- CossedduS.; InfanteI.Force Field Parametrization of Colloidal CdSe Nanocrystals Using an Adaptive Rate Monte Carlo Optimization Algorithm. J. Chem. Theory Comput.2017, 13 ( (1), ), 297–3082806877610.1021/acs.jctc.6b01089.^1^*This article gives a procedure to obtain highly accurate and transferable classical force field parameters to describe the interactions in nanocrystal–ligand–solvent systems, thus allowing classical molecular dynamics simulations to be performed with realistic nanocrystal sizes and ligands and explicit solvent molecules, paving the way toward an atomistic understanding of the dynamical nanocrystal surface region.*
- ZapataF.; RidderL.; HiddingJ.; JacobC. R.; InfanteI.; VisscherL.QMflows: A Tool Kit for Interoperable Parallel Workflows in Quantum Chemistry. J. Chem. Inf. Model.2019, 59 ( (7), ), 3191–31973126029210.1021/acs.jcim.9b00384PMC6651270.^2^*This paper describes an open-source Python-based platform that efficiently executes composite workflows to carry out a large number of time-consuming molecular simulations with complicated dependencies.*
- DrijversE.; De RooJ.; MartinsJ. C.; InfanteI.; HensZ.Ligand Displacement Exposes Binding Site Heterogeneity on CdSe Nanocrystal Surfaces. Chem. Mater.2018, 30 ( (3), ), 1178–1186.^3^*This article reports a thermodynamic analysis of the displacement equilibrium of different amine ligands from a given binding site at the CdSe nanocrystal surface, with a particular focus on the enthalpic and entropic contributions.*
- De NolfK.; CossedduS. M.; JasieniakJ. J.; DrijversE.; MartinsJ. C.; InfanteI.; HensZ.Binding and Packing in Two-Component Colloidal Quantum Dot Ligand Shells: Linear versus Branched Carboxylates. J. Am. Chem. Soc.2017, 139 ( (9), ), 3456–34642823447410.1021/jacs.6b11328.^4^*This paper describes a ligand exchange procedure to introduce entropic ligands at the CdSe nanocrystal surface and pioneers the use of molecular dynamics simulations to elucidate the effects of this replacement on the dynamical behavior of the ligand shell and in particular on the ligand distribution.*

## Introduction

**Figure 1:**
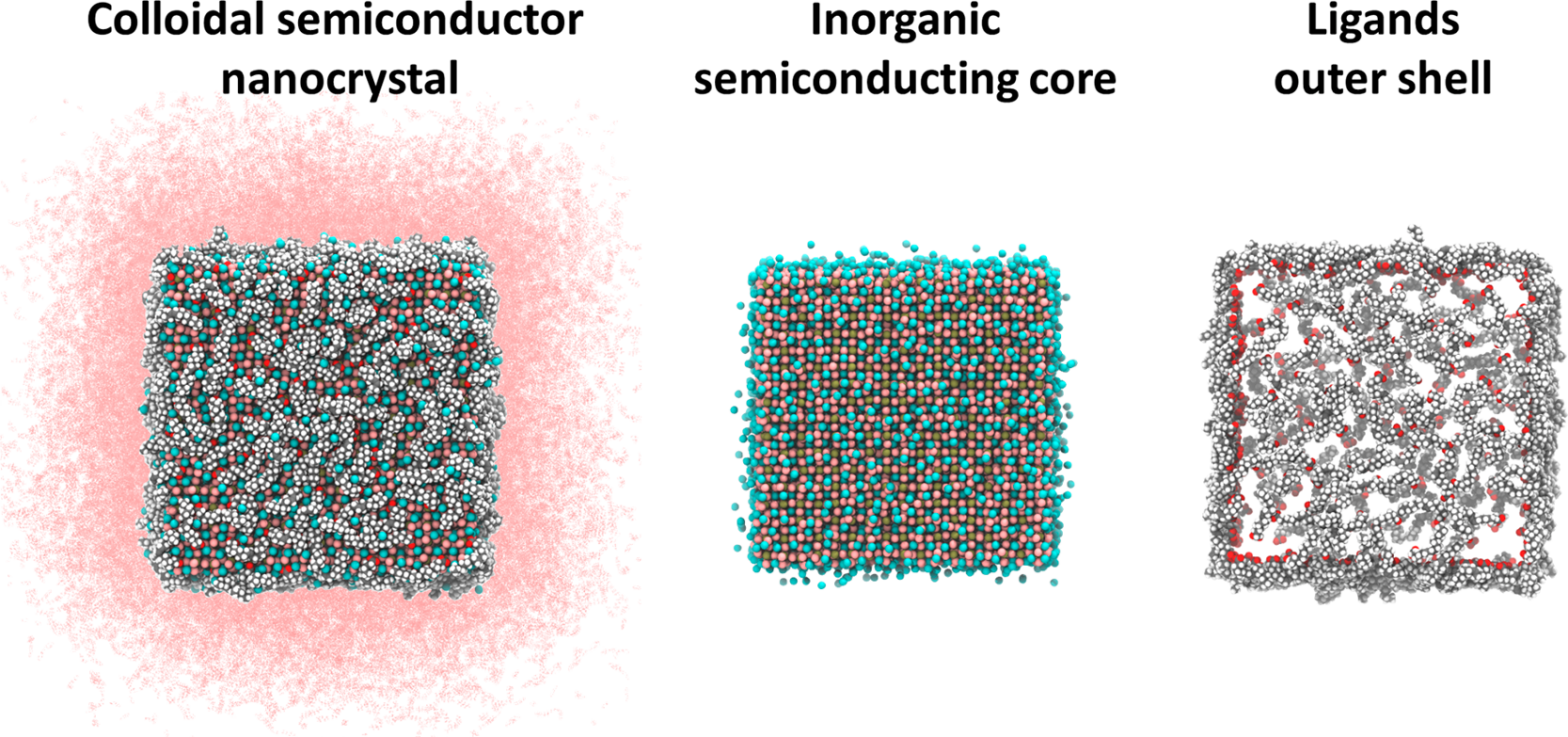
Stick and ball representations of (left) a colloidal perovskite nanocrystal dissolved in octadecene, composed of (center) a 6 nm-sided cubic CsPbBr_3_ core and (right) an outer shell of oleate ligands. The color code used for the atoms is Cs, cyan; Pb, orange; Br, pink; O, red; C, gray; H, white; the solvent is represented by pink dots.

Colloidal semiconductor nanocrystals (NCs), also called quantum dots, can be regarded as hybrid inorganic–organic materials consisting of a nanosized inorganic semiconducting core surrounded by an outer shell of organic ligands, as illustrated in Figure 1. The NC inorganic core is the optically active part and plays a role in defining the optoelectronic characteristics of the overall material, while the primary function of the ligand shell is to stabilize the NC in several types of organic solvents.

Surface ligands, however, are essential in several other aspects of the colloidal nanocrystal chemistry: (1) at high surface coverages, they suppress nonradiative recombination centers (defects);^5−9^ (2) in conventional II–VI and IV–VI nanocrystals, they can shift the energetic position of the conduction and valence band edges,^5,10−12^ a feature that is still to be proven for other semiconductor nanocrystals; and (3) they can be exchanged with other types of ligands, in particular with shorter ligands that can act as electronic bridges between NCs, for example to improve electron–hole extraction when the solution is cast into films.^5,13−15^ This last step is crucial in engineering and optimizing efficient photoactivated conductive devices. From this set of characteristics, it becomes clear that the surface chemistry of NCs, if deeply understood, can be used as a platform for the rational design of ligands, which in our view represent the most critical feature of colloidal NCs.

Unsurprisingly, the successful synthesis of a given NC material is invariably followed by a plethora of experimental reports that propose new surface ligands to improve the nanocrystals’ stability and enhance their optoelectronic properties.^16−18^ These experimental attempts usually employ the candidate ligands either in postsynthesis ligand exchange procedures or directly during the synthesis of the NCs. To date, the search for optimal ligands is mostly based on an inefficient empirical approach, in which the number of syntheses can grow drastically before a ligand that fits perfectly with the NC core surface is identified. On the positive side, this problem can nowadays be tackled atomistically by carrying out classical molecular dynamics (MD) simulations on NC–ligand–solvent systems with realistic NC sizes and ligands and explicit solvent molecules, thus providing an atomistic picture of the dynamical nanocrystal surface region. With these recently developed tools in hand, our group is planning to investigate the role of parameters such as ligand entropy, ligand–ligand steric hindrance, and ligand–core interactions in the processes of ligand binding/displacement and NC dissolution/precipitation, ultimately providing a strategy to maximize the surface coverage and boost both the colloidal stability and efficiency of the NCs. Unfortunately, we are also aware that this type of study, which is already by itself very challenging from a computational standpoint, allows the exploration of only a limited part of the ligand chemical space. To put things in perspective, according to the Generated DataBase, also called GDB-17, created by Reymond et al. in the drug discovery field, the total number of possible organic ligands with up to 17 atoms (excluding hydrogens) that satisfy simple chemical and synthesis rules is in fact 166 billion.^19^ This is about 3 orders of magnitude larger than the number of ligands that have been synthesized to date, which is about 169 million structures according to the CAS Registry.^20^ This hints at the idea that a clear help in the rationalization and minimization of the synthetic effort can be provided by following the example set in the drug discovery field, where the search for the best drugs nowadays follows an efficient integration of computational chemistry tools and machine learning models. We believe that finding a pathway to compute rapidly ligand properties that best describe ligands at the NC surface is a strategy that can be successful in the near future. We can expect that when a sufficiently trained data set of ligand properties becomes sufficiently large for machine learning algorithms to be predictive, this high-throughput approach will be able to suggest optimal ligand candidates to be assessed in the experiments.

## Where Atomistic Calculations Stand as of Today

In recent years, our group has developed automated tools to obtain highly accurate force field parameters for nanocrystal–ligand–solvent systems.^1,21^ In practice, we are able to describe with chemical accuracy the interactions between the inorganic nanocrystal core and surface ligands as well as those within the nanocrystal core through a simple combination of Lennard-Jones potentials^22,23^ and Coulombic electrostatic interactions. The unknown parameters (the depth of the potential well as the ε parameter, the size of the ion as the σ parameter, and the atomic charges) are fitted against density functional theory^24^ (DFT) reference data, ultimately attaining a DFT-quality description of the overall system. Because of the good transferability of the parameters to large sizes and the possibility of also including the solvent explicitly, we are now able to perform classical MD simulations for realistic NC–ligand–solvent interfaces with up to 1 million atoms in the simulation box.^4^ This opens the way, for the first time, to the possibility of using MD methodologies that have mostly been applied in biochemistry and drug discovery also in the colloidal nanochemistry field. Classical MD simulations can be used to study NC–ligand–solvent systems featuring different surface coverages to unveil the dynamical behavior of the ligands at the nanocrystal surface, revealing for example how ligands (i) cluster via dispersive (i.e., non-covalent) forces or prefer a random distribution, (ii) diffuse at the surface, and (iii) undergo attachment and displacement events. These atomistic studies can be performed by taking advantage of the abundance of analysis tools offered by classical MD simulation packages.^25−27^ Most importantly, such packages usually also enable calculation of the free energy of complexation of ligands to a receptor and the free energy of solvation of ligands in a solvent, features that are particularly attractive to us in evaluating the relevant energetic terms for the main chemical processes occurring at the nanocrystal surface.

To obtain a comprehensive picture of the NC surface chemistry, one could aim at computing these factors for a set of ligands that intuitively spans the chemical space of molecular surfactants. Unfortunately, although this approach would be very valuable in obtaining fundamental insights into NC surface chemistry, it has the drawback of being computationally demanding. If we wish to explore a much larger set of ligands, our main task will boil down to finding and computing specific descriptors, i.e., chemico-physical properties of the ligands that obey specific rules of thumb that we will define later in the text. The best approach to compute these descriptors will likely require a better understanding of the NC surface chemistry, although we expect that they will be computed either by using first-principles calculations, like DFT,^24^ or by defining features that can be straightforwardly computed, like the volume occupied by the ligands, the surface polar area, the synthesizability score, etc. Irrespective of the methodology employed to compute these properties, it is expected that a massive number of calculations must be performed, and it is thus paramount that such calculations are carried out seamlessly using quantum chemistry software packages and algorithms that are fast, efficient, and well-integrated one with another. For this purpose, we have recently developed QMflows,^2^ a Python-based library that seamlessly executes complex workflows consisting of a large number of calculations with complicated dependencies. It also allows the processing of an enormous number of inputs/outputs and postprocessing data collection and analysis. Ultimately, we will be able to write a series of composite workflows that will enable us to compute a vast number of ligand and ligand-NC related properties, store them in databases, and finally process them with machine learning algorithms.

## Revisiting the Chemical Equilibria at NC Surfaces

**Figure 2:**
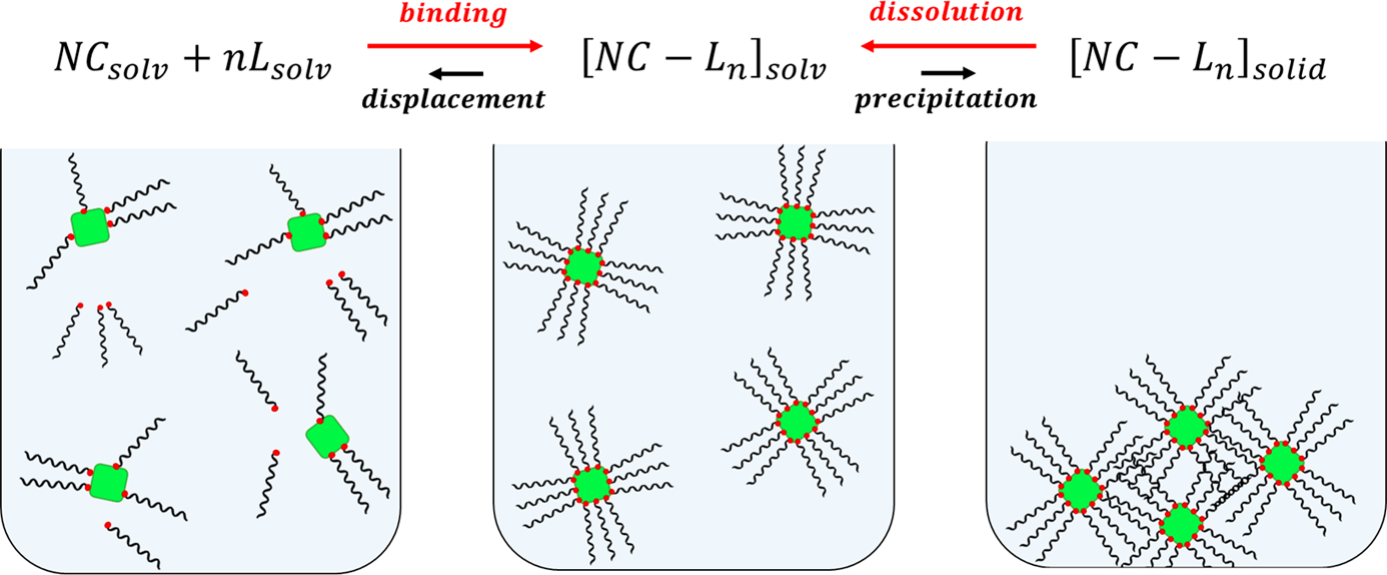
Schematic representation of a comprehensive framework that includes (left) the binding/displacement of ligands at the nanocrystal surface in solution and (right) the precipitation/dissolution of nanocrystal–ligand complexes in organic solvents. Ideally, the first equilibrium should be pushed to the right and the second equilibrium to the left, toward the fully passivated nanocrystals in solution.

To address the above challenges atomistically and set the base for a comprehensive description the NC surface chemistry, it is paramount for us to formalize the mechanism of ligand binding/displacement and NC dissolution/precipitation in a unique thermodynamic framework that is suitable to be addressed with atomistic calculations (Figure 2).

In this respect, we start from the work of Peng et al.,^28^ who demonstrated that identifying the factors that govern the dissolution/precipitation equilibrium of nanocrystal–ligand complexes in organic solvents by means of a quantitative thermodynamic model enables the design of surface ligands that maximize the NCs’ solubility, thus mastering the processability challenge of colloidal nanocrystals. In particular, their thermodynamic analysis revealed the decisive role of enthalpy and entropy changes during dissolution of nanocrystals coated with *n*-alkanoate ligands, namely, (i) a large enthalpy cost for dismantling the strong ligand–ligand interdigitation between adjacent particles during the precipitation step and (ii) a massive increase in intramolecular entropy related to the ligands’ C–C σ-bond rotation and skeletal bending in solution. Subsequently, Peng et al.^29^ introduced the so-called “entropic ligands” concept to interrupt crystalline ligand–ligand packing in the solid but harvest conformational entropy in solution: for example, they demonstrated an increase in the solubility of various nanocrystals by several orders of magnitude by just replacing *n*-alkanoate ligands with irregularly branched ones.

Although this aspect is crucial to improve the solubility of colloidal nanocrystals, there is a second and perhaps even more important challenge for colloidal nanocrystal chemistry, which is to achieve complete passivation of the NC surface by ligands to maximize the surface coverage, a key aspect for the elimination of surface defects and for the improvement of the NCs’ optoelectronic characteristics.

**Figure 3:**
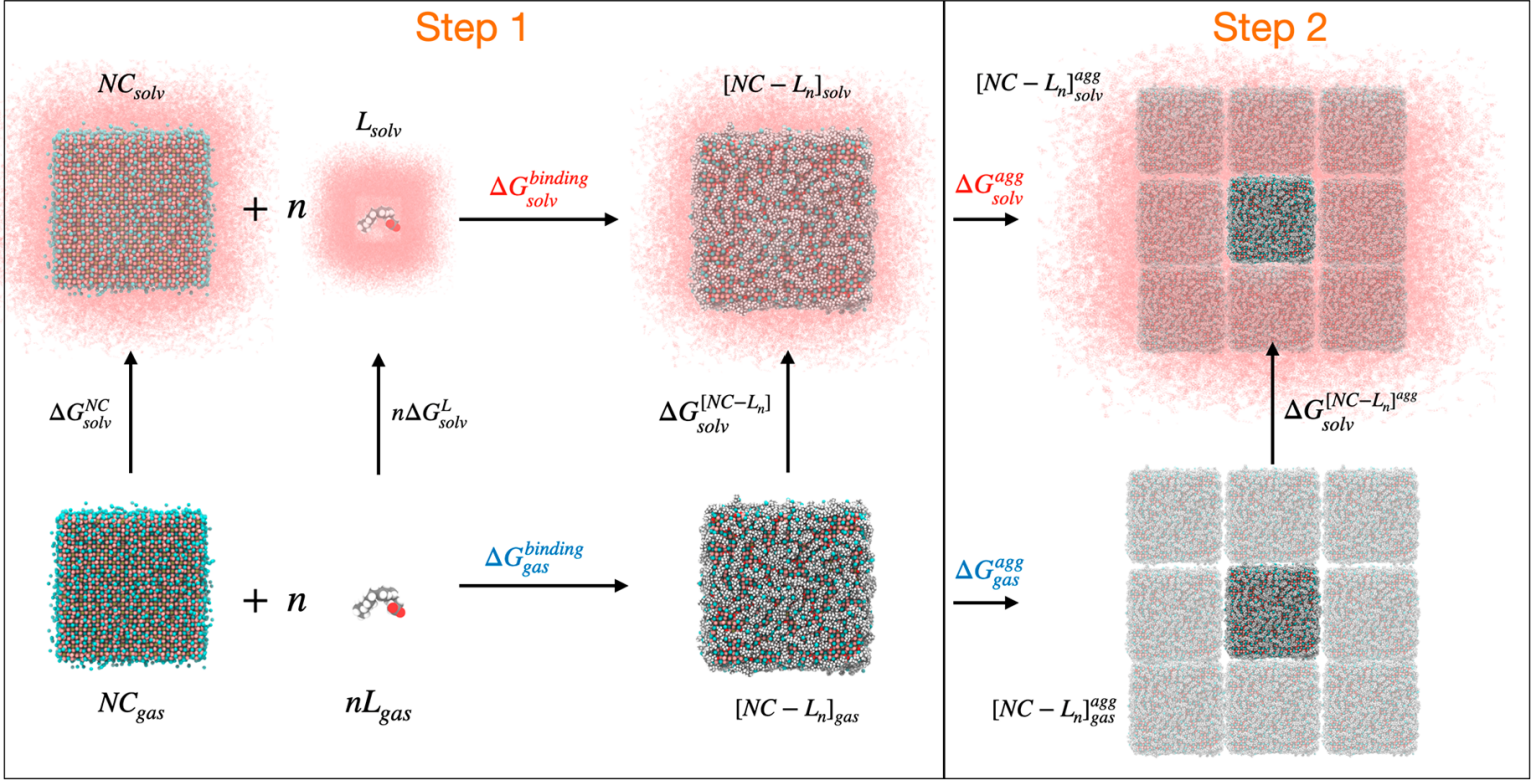
Thermodynamic cycle for the binding interaction between the ligands and the nanocrystal and for the agglomeration (precipitation) of nanocrystal–ligand complexes in a given solvent.

As anticipated at the beginning of this section, it is thus critical to place on the same ground the process of binding/displacement of ligands at the nanocrystal surface in solution and the process of precipitation/dissolution of nanocrystal–ligand complexes in organic solvents (see Figure 2). In this picture, ideal ligands should favor the intermediate state by simultaneously pushing the first equilibrium to the right (maximizing the surface coverage) and the second equilibrium to the left (maximizing the solubility, similarly to the entropic ligands). For practical computational purposes, we propose the thermodynamic cycle sketched in Figure 3 that transfers the most critical processes from the solvent to the gas phase, thus providing a substantial computational advantage.

In this cycle, NC is the unpassivated nanocrystal, L is the ligand, and [NC–L_*n*_] is the NC capped with *n* surface ligands L. According to the thermodynamic cycle above, we can express the total free energy of the process from the separate fragments to NC precipitation as
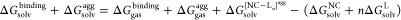
1

Since our aim is to shift the equilibrium toward the fully passivated nanocrystals in solution, the free energy of the ligand binding, Δ*G*_solv_^binding^, should be minimized, i.e. more exergonic, while that of NC agglomeration, Δ*G*_solv_^agg^, should be maximized, i.e. more endergonic. For practical purposes, however, it is more convenient to study the two processes separately, as will be done from now on. It should be noted that in typical apolar solvents, the ligands are not attached or displaced from the nanocrystal surface alone but as neutral ion pairs or as a Z-type ligand, composed of the anionic/cationic ligand bound to a metal ion/anion from the NC, respectively. The symbol L, employed for clarity, actually represents the overall ion pair, thus ensuring the consistency of the binding process under examination with the chemistry of precursors used in the NC synthesis.

### Step 1: Binding/Displacement of Ligands at the NC Surface

As sketched in Figure 3, the separate fragments (the bare NC and the *n* ligands L that are finally present at its surface) are considered as the starting point in the binding process, thus immediately and explicitly taking into account the expected dependence of the binding free energy on the surface coverage, expressed as the fraction of surface sites occupied by ligands. In this framework, the ligand binding process is described as
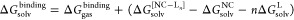
2where we can group the terms in parentheses by defining a solvation energy term ΔΔ*G*_solv_ that represents the gain/loss in free energy obtained by solvating the overall [NC–L_*n*_] system compared with the separate components at infinite dilution:
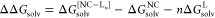
3

**Scheme 1:**
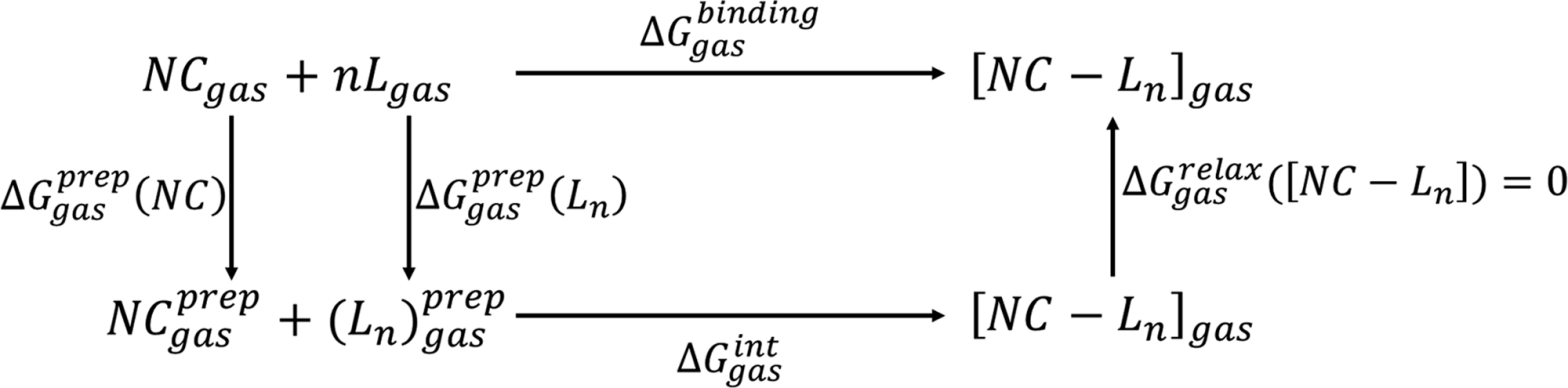
Thermodynamic Cycle for the Binding Interaction between the Ligands and the NC in the Gas Phase

The next term to be discussed in detail is the total binding free energy of surface ligands to the generic nanocrystal in the gas phase, Δ*G*_gas_^binding^. As in many quantum-mechanical approaches,^30^ we can conveniently decompose the Δ*G*_gas_^binding^ term as the following sum:
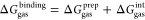
4The first term of this decomposition, Δ*G*_gas_^prep^, is the preparation (deformation) energy to bring the starting fragments NC and L from their configuration at infinite distance from each other to the configuration they assume in the final [NC–L_*n*_] system. The second term of the sum, Δ*G*_gas_^int^, represents the local interaction and relaxation energy between the as-prepared fragments. A detailed view of the ligand binding process in the gas phase using this approach is sketched in Scheme 1.

We can expect the ligands to undergo a large reorganization, both enthalpic and entropic, from the starting configuration to that found at the NC surface,^3^ similar to what was observed by Peng et al. during the precipitation of nanocrystal–ligand complexes in organic solvents. In other words, we can expect the Δ*G*_gas_^prep^(L_*n*_) term, corresponding to the ligand preparation energy, to be very large. On the other hand, we can assume that the core of the NC remains substantially unchanged during the preparation process, meaning that Δ*G*_gas_^prep^(NC) ≈ 0:
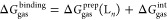
5Here we can further decompose the first term to capture the enthalpic and entropic contributions in the reorganization of ligands at the NC surface, thus obtaining:
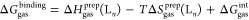
6

The free binding energy for binding of surface ligands to a generic NC in a given solvent can ultimately be expressed as
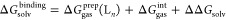
7or equivalently as
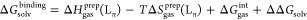
8Importantly, we can expect that for a given ligand, the binding free energy is the largest in absolute value (i.e., most exergonic) at a certain surface coverage, *θ*_c_, which is simply defined by the formula
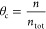
9where *n* is the number of surface sites effectively occupied by the ligands at a given surface coverage and *n*_tot_ is the total of available surface sites. In our picture, the minimum of free energy, obtained at the equilibrium value of the surface coverage, accounts for the dependence of all of the terms in eq 8 on *θ*_c_. We can highlight this dependence by rewriting eq 8 as
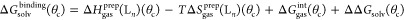
10Each term in eq 10 has an intuitive and relevant chemical meaning:

- Δ*G*_gas_^int^(θ_c_) is the actual interaction between the NC and the surface ligands L (or, more precisely, between the NC and the metal–ligand or anion–ligand ion pairs), and it is always exergonic (i.e., Δ*G*_gas_^int^(θ_c_) is negative). It mostly depends on the particular interaction between the anchoring group of the ligand and the NC, and thus, we expect its strength to widely fluctuate according to the type of NC material considered and the type of functional group anchored to the surface (−COO, −S, −O, −PO(OH)O, etc.).
- Δ*H*_gas_^prep^(L_*n*_)(θ_c_) is an enthalpic term that evaluates the agglomeration interaction among the ligands, and its sign is strongly dependent on the surface coverage. At some coverages, ligands can favorably pack through weak dispersive ligand–ligand bonding interactions (i.e., Δ*H*_gas_^prep^(L_*n*_)(θ_c_) is negative). At high coverages, however, the repulsive steric interactions (i.e., Δ*H*_gas_^prep^(L_*n*_)(θ_c_) is positive) would probably dominate.^4^
- Δ*S*_gas_^prep^(L_*n*_)(θ_c_) is the entropic term and is reminiscent of the entropic ligand definition by Peng et al. It favors ligands as detached from the surface, and thus, it is always negative, making the −*T*Δ*S*_gas_^prep^(L_*n*_) energy term positive. This is always true, as the number of configurations that ligands can assume is much higher at infinite dilution than on the surface, where the translational, rotational, and conformational mobility is reduced. At maximum packing, the entropic term is likely to be at its maximum because we can expect the ligand mobility to be at its minimum.
- ΔΔ*G*_solv_(θ_c_) = Δ*G*_solv_^[NC–L_*n*_]^(θ_c_) – Δ*G*_solv_^NC^ – *n*Δ*G*_solv_^L^ is the gain/loss in free energy resulting from solvation of the system relative to its components at infinite dilution. It is possible that the ligand-passivated NC presents a solubility/solvation free energy that is larger than the sum of those for its components, similar to what reported for micelles. This aspect is still unknown and has not been explored before, either computationally or experimentally. Moreover, the sign of this term is expected to vary strongly with the type of solvent.

**Figure 4:**
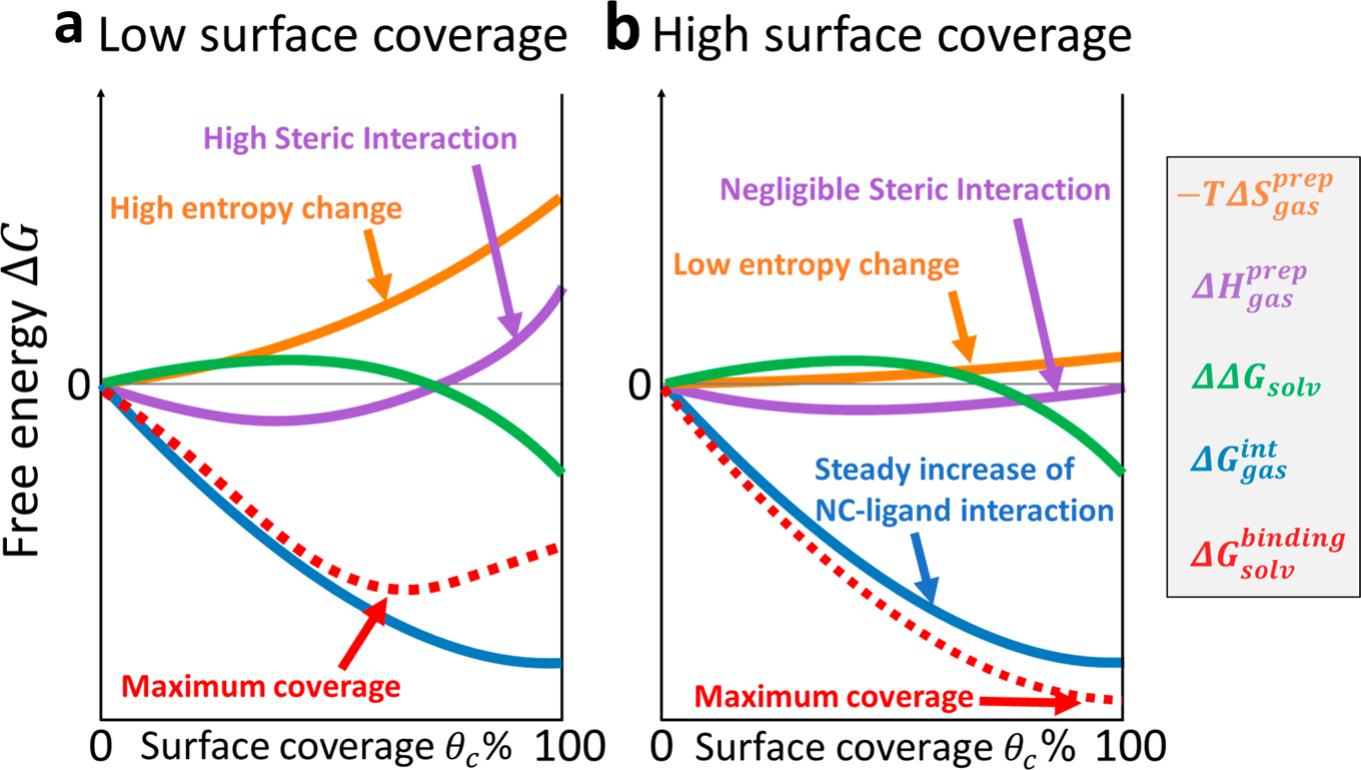
Expected functional dependence of each term in eq 10 on the surface coverage *θ*_c_ for two types of ligands. (a) A low surface coverage at equilibrium is expected from bulky ligands (high steric hindrance at the NC surface) featuring a high entropic change from the NC surface to the solution. (b) A high surface coverage (corresponding to a minimized binding free energy) might be obtained with small ligands that avoid steric interactions and have low entropy changes.

For a given ligand, the total binding free energy can be determined as the sum of the contributions of all of the above factors, which act differently as functions of the surface coverage (dashed red line in Figure 4a). Ideally, ligands will perform best when the surface coverage is near unity because all of the surface vacancies are expected to be passivated (surface traps suppressed) (Figure 4b). Even though a rigorous quantitative analysis is needed, we can intuitively expect the best ligands to match the following characteristics: (1) they have a functional group that binds strongly to the NC surface; (2) they are small enough to avoid steric hindrance at the NC surface; (3) they favor interligand packing via weak (e.g., van der Waals) bonding interactions at the NC surface; (4) they have low entropy changes; and (5) they are soluble enough in the solvents used in the synthesis to stabilize the colloidal dispersion of nanocrystals.

### Step 2. Precipitation/Dissolution Equilibrium of NCs

The ligand binding model constructed in the previous section already helps in defining some rules of thumb to identify surface ligands that fit best to a given NC material, i.e., that maximize the surface coverage by pushing the binding/displacement equilibrium of Figure 3 toward the passivated NC in solution. We will now consider the precipitation/dissolution equilibrium, which should be oriented toward dissolution of the NCs by maximizing the free energy of agglomeration. According to Figure 3, the latter can be written as
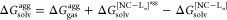
11Here we can use the following approximations: Δ*G*_solv_^[NC–L_*n*_]^agg^^ ≈ 0 and Δ*G*_gas_^agg^ = *n*_facet_Δ*G*_gas_^facet–facet^. The first one is justified by the fact that, as a first approximation, the precipitated nanocrystal–ligand complex interacts locally only with its nearest neighbors, with the solvent playing only a negligible role. This is key to simplify the interaction of the NC with its surroundings to an effective one-body problem, although more sophisticated models of the agglomerate should also include the presence of solvent trapped between nanocrystals. The second approximation stems from the idea that the interactions between [NC–L_*n*_] systems in the solid state preferentially occur via facet-to-facet interactions. Although this is better justified during the formation of superlattices, we can practically assume that something similar takes place during the precipitation of NCs. It should be noted that we are attempting to simplify a complex problem to make it tractable for computational purposes. On the basis of these arguments, the agglomeration term becomes
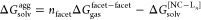
12In the first term, Δ*G*_gas_^facet–facet^ represents the local pair interaction between a given facet of the [NC–L_*n*_] system and the adjacent facet of one of its nearest neighbors in the precipitate. We can further decompose Δ*G*_gas_^facet–facet^ in terms of ligand–ligand interactions, as expressed by the term Δ*G*_gas_^L_(*m*)_–L_(*m*)_^, which corresponds to free energy for the interaction of the *m* ligands on a facet (*m* = *n*/*n*_facet_) with the *m* ligands on the adjacent facet. Although the theoretical model proposed by Peng et al. basically neglects the NCs’ core–core interactions in the solid state, the experimental measurements presented in the same paper highlight the importance of this term in the dissolution enthalpy of nanocrystal–ligand complexes. On the basis of this evidence, we take this interaction term into account. On the other hand, we can assume that the interaction between the core of a NC and the ligands of the neighboring facet in the precipitate is negligible. With these considerations, the facet-to-facet pair interaction in the solid state can be ultimately expressed as
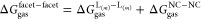
13where Δ*G*_gas_^NC–NC^ is the nanocrystal-to-nanocrystal local interaction. Here again, we can further decompose this expression to capture the enthalpic and entropic contributions to the precipitation of the NCs, thus obtaining
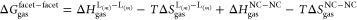
14The free energy of precipitation for a generic [NC–L_*n*_] complex in a given solvent in eq 12 can now be equivalently expressed as
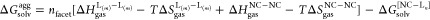
15

As in the case of the ligand binding free energy, we can expect each term in eq 15 to be strongly dependent on the surface coverage *θ*_c_. Consequently, the largest (i.e., most endergonic) value of the agglomeration free energy, resulting in the maximal solubility of nanocrystal–ligand complexes, should be obtained at a certain surface coverage. We can evidence this dependence by rewriting eq 15 as
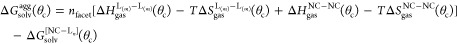
16Each term in this formula has also an intuitive chemical meaning, and we can expect the following dependences on *θ*_c_:

- Δ*H*_gas_^L_(*m*)_–L_(*m*)_^(*θ*_c_) is the enthalpic term that represents the facet-to-facet agglomeration interaction among the ligands in the precipitate and should be strongly dependent on the surface coverage. Below a certain coverage, the ligand spacing allows favorable interdigitation through nonbonding ligand–ligand interactions (i.e., Δ*H*_gas_^L_(*m*)_–L_(*m*)_^(*θ*_c_) is negative). At high coverages, depending on the ligand bulkiness, repulsive steric interactions are expected to have a growing importance (i.e., Δ*H*_gas_^L_(*m*)_–L_(*m*)_^(*θ*_c_) is positive).
- Δ*S*_gas_^L_(*m*)_–L_(*m*)_^(*θ*_c_) is the entropic term that is maximized by Peng et al. to design the entropic ligands. This term is always negative (and consequently, the −*T*Δ*S*_gas_^L_(*m*)_–L_(*m*)_^(*θ*_c_) energy term is positive) since the number of conformational degrees of freedom drastically decreases during precipitation of the [NC–L_*n*_] system. The variation of the entropy of the ligands in going from isolated to precipitated NCs is dependent on the capacity of the ligands to effectively pack in the solid state, so it should reach a plateau beyond a certain surface coverage.
- Δ*H*_gas_^NC–NC^(*θ*_c_) is the enthalpic term that describes the core–core interactions in the precipitate. This agglomeration term should always be negative and is expected to dominate at low surface coverage (corresponding to solid-state packing between NCs), where it acts as a driving force for precipitation of the nanocrystals.
- Δ*S*_gas_^NC–NC^(*θ*_c_) is the entropic term that accounts for the locking of rotational and translational degrees of freedom of the nanocrystal core during agglomeration of [NC–L_*n*_] complexes. This term is always negative, and therefore, the −*T*Δ*S*_gas_^NC–NC^(*θ*_c_) energy term in eq 16 is positive. We can expect this entropy variation to be almost independent of the surface coverage.
- Δ*G*_solv_^[NC–L_*n*_]^(*θ*_c_) is the free energy of solvation for ligand-passivated NCs, which was already encountered in the previous section. Here again, the behavior of this term with the surface coverage is difficult to predict and should widely fluctuate with the type of solvent.

**Figure 5:**
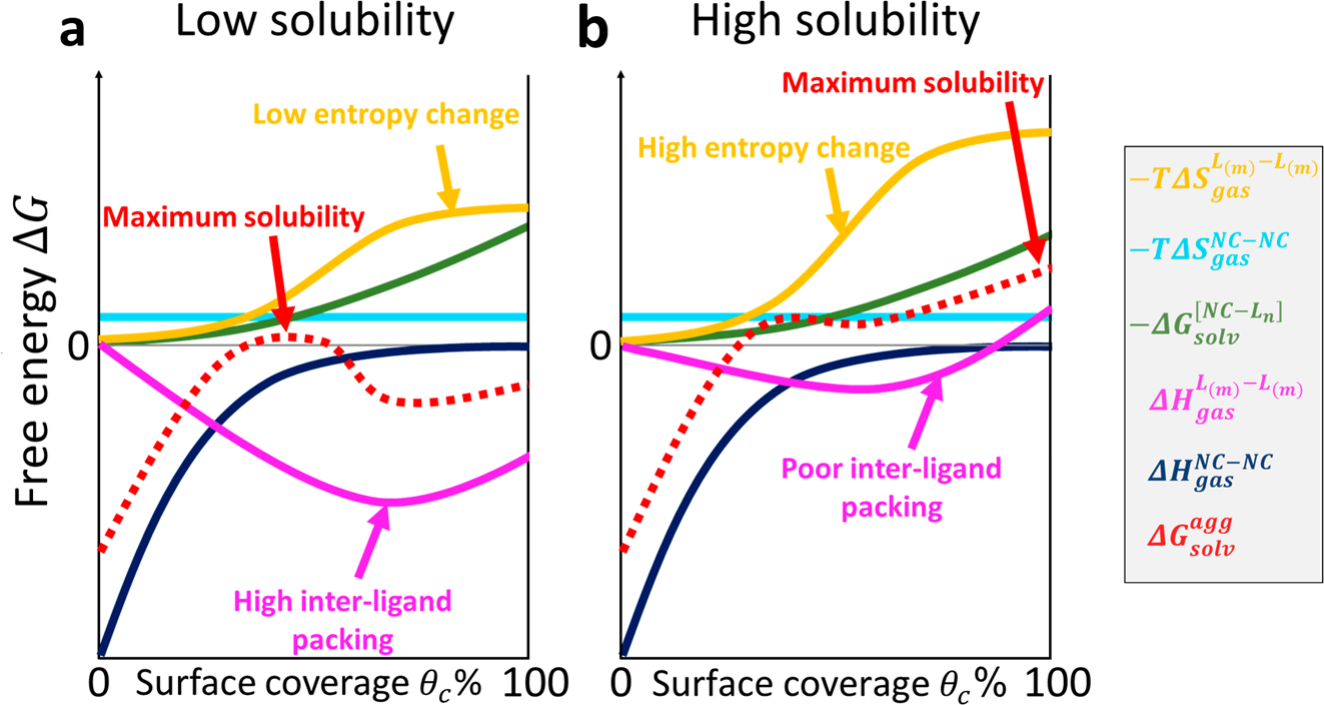
Expected functional dependence of each term in eq 16 on the surface coverage *θ*_c_ for two types of ligands. (a) Maximum solubility is expected at low surface coverages for ligands that favorably interdigitate in the solid state and feature a low conformal entropy in solution. (b) Maximum solubility might be obtained at high coverages with bulky ligands that avoid ligand–ligand packing in the precipitate and have a high conformational entropy in solution.

For a given ligand–nanocrystal–solvent combination, the total free energy of agglomeration of the nanocrystal–ligand complexes can be obtained simply by adding the contributions of all of the above factors and, like its component terms, is a function of the surface coverage (dashed red line in Figure 5a). In our framework, the entropic ligands defined by Peng et al. can now be seen as the ligands that are able to maximize the free energy of precipitation, thus favoring the dissolution process and boosting the NCs’ solubility. In fact, Peng et al. recommended minimization of the interligand packing for destruction of the crystalline chain–chain interactions in solids and maximization of the ligands’ intramolecular entropy (Figure 5b) as a universal strategy to battle the processability challenge of colloidal nanocrystals.

Finally, it is important to notice that mixing of different types of passivating ligands at the nanocrystal surface has also been successfully used to enhance both the nanocrystal optoelectronic properties and solubility. For example, Peng et al. reported an increase of several orders of magnitude in the solubility of CdSe nanocrystals by simple addition of a short-chain ligand (hexanoate) to a pure shell of a long-chain ligand (myristate).^31^ Similarly, two-component ligand shells consisting of primary alkylamines with moderate chain lengths mixed with long-chain oleate ligands have been employed by Quarta et al. to obtain colloidal CsPbBr_3_ NCs characterized by effective surface passivation and, as a consequence, enhanced optical properties.^32^ The search for a multicomponent ligand shell that matches, as a whole, the above characteristics represents an interesting and flexible alternative to the search for single ideal ligands and should be included in the above thermodynamic framework in the future.

## A Bright Future for Engineering of Surface Ligands

At this point, we have a comprehensive picture in which all of the terms in the thermodynamic cycle of Figure 3 can be calculated with standard classical and quantum chemistry tools.

Unfortunately, some features that help the ligands bind to the NC surface (proposed in step 1) are in sharp contrast with the definition of entropic ligands proposed by Peng et al. to maximize the solubility of the capped NCs in organic solvents. To better explain this apparent contradiction, we can look back at the unique equilibrium that includes both binding/displacement of ligands at the NC surface in solution and precipitation/dissolution of nanocrystal–ligand complexes, as illustrated in Figure 2. In this unified picture, promoting the ligand–ligand packing at the NC surface helps to maximize the surface coverage (first equilibrium pushed to the right) but could also favor crystalline ligand–ligand interdigitation in the precipitate, thus dramatically decreasing the NC solubility. Similarly, ligands with a huge conformational entropy (“entropic ligands”) greatly stabilize the colloidal dispersion (second equilibrium pushed to the left) but could assist the displacement of ligands from the NC surface, thus deteriorating their optoelectronic properties by introducing surface defects.

**Figure 6:**
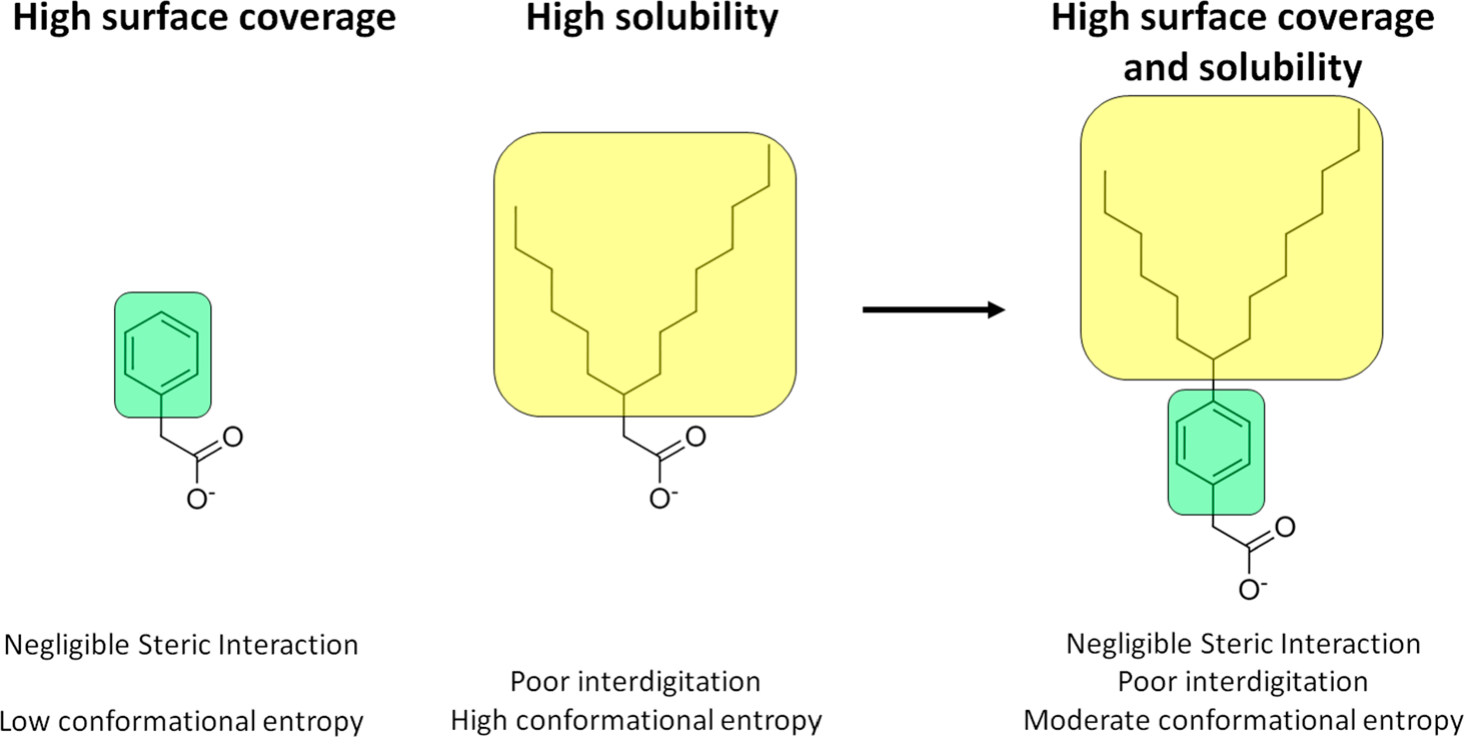
Example of a ligand that combines favorable packing at the NC surface with prevention of interdigitation in the solid state.

Overall, ideal ligands are those that favor the intermediate state, maximizing both NC surface coverage (efficiency) and NC solubility. We should thus balance our rules of thumb for the rational design of surface ligands accordingly in order to take into account both of these critical processes. The *ligand anchoring group* plays a critical role in minimizing the ligand binding energy and should ensure that the binding strength to the NC surface is as high as possible. The *ligand backbone* should be engineered in order to (1) favor ligand–ligand packing at the NC surface (i.e., in its initial segment, close to the anchoring group), (2) prevent ligand–ligand interdigitation between nanocrystal–ligand complexes (e.g., by adding some branching alkylic chains in its terminal segment), (3) have a balanced entropy change (especially in the case of weak binding), and (4) be soluble enough in the solvents used in the synthesis to stabilize the colloidal dispersion of nanocrystals. An example of a possible candidate is sketched in Figure 6.

In reality, an enormous number of structures (ligands) can be considered and characterized to find candidate ligands with these finely tuned properties. Thus, here we arrive at the future challenges of this field, which includes the application of advanced statistical analysis tools like machine learning. The identification of optimal ligands that demonstrate improved NC optoelectronic qualities (photoluminescence, color purity, etc.) and processability will indeed require the construction of a large data set of computed ligand properties. The most demanding task will be to build a robust training set of thousands of structures in a practical amount of time, which will boil down to being able to compute and store the properties of interest for each ligand in a very fast and efficient way. As pointed out at the beginning of this Account, this challenge is now within reach thanks to the set of computational tools that our group has recently developed.

## References

[ref1] Cosseddu S.; Infante I. Force Field Parametrization of Colloidal CdSe Nanocrystals Using an Adaptive Rate Monte Carlo Optimization Algorithm. J. Chem. Theory Comput. 2017, 13, 297–308. 10.1021/acs.jctc.6b01089. 28068776

[ref2] Zapata F.; Ridder L.; Hidding J.; Jacob C. R.; Infante I.; Visscher L. QMflows: A Tool Kit for Interoperable Parallel Workflows in Quantum Chemistry. J. Chem. Inf. Model. 2019, 59, 3191–3197. 10.1021/acs.jcim.9b00384. 31260292 PMC6651270

[ref3] Drijvers E.; De Roo J.; Martins J. C.; Infante I.; Hens Z. Ligand Displacement Exposes Binding Site Heterogeneity on CdSe Nanocrystal Surfaces. Chem. Mater. 2018, 30, 1178–1186. 10.1021/acs.chemmater.7b05362.

[ref4] De Nolf K.; Cosseddu S. M.; Jasieniak J. J.; Drijvers E.; Martins J. C.; Infante I.; Hens Z. Binding and Packing in Two-Component Colloidal Quantum Dot Ligand Shells: Linear versus Branched Carboxylates. J. Am. Chem. Soc. 2017, 139, 3456–3464. 10.1021/jacs.6b11328. 28234474

[ref5] Boles M. A.; Ling D.; Hyeon T.; Talapin D. V. The Surface Science of Nanocrystals. Nat. Mater. 2016, 15, 141–153. 10.1038/nmat4526. 26796733

[ref6] Almeida G.; Infante I.; Manna L. Resurfacing Halide Perovskite Nanocrystals. Science 2019, 364, 833–834. 10.1126/science.aax5825. 31147510

[ref7] Giansante C.; Infante I. Surface Traps in Colloidal Quantum Dots: A Combined Experimental and Theoretical Perspective. J. Phys. Chem. Lett. 2017, 8, 5209–5215. 10.1021/acs.jpclett.7b02193. 28972763 PMC5651579

[ref8] Houtepen A. J.; Hens Z.; Owen J. S.; Infante I. On the Origin of Surface Traps in Colloidal II-VI Semiconductor Nanocrystals. Chem. Mater. 2017, 29, 752–761. 10.1021/acs.chemmater.6b04648.

[ref9] Bodnarchuk M. I.; Boehme S. C.; Ten Brinck S.; Bernasconi C.; Shynkarenko Y.; Krieg F.; Widmer R.; Aeschlimann B.; Günther D.; Kovalenko M. V.; Infante I. Rationalizing and Controlling the Surface Structure and Electronic Passivation of Cesium Lead Halide Nanocrystals. ACS Energy Lett. 2019, 4, 63–74. 10.1021/acsenergylett.8b01669. 30662955 PMC6333230

[ref10] Giansante C. Surface Chemistry Control of Colloidal Quantum Dot Band Gap. J. Phys. Chem. C 2018, 122, 18110–18116. 10.1021/acs.jpcc.8b05124.

[ref11] Frederick M. T.; Weiss E. A. Relaxation of Exciton Confinement in CdSe Quantum Dots by Modification with a Conjugated Dithiocarbamate Ligand. ACS Nano 2010, 4, 3195–3200. 10.1021/nn1007435. 20503978

[ref12] Brown P. R.; Kim D.; Lunt R. R.; Zhao N.; Bawendi M. G.; Grossman J. C.; Bulović V. Energy Level Modification in Lead Sulfide Quantum Dot Thin Films through Ligand Exchange. ACS Nano 2014, 8, 5863–5872. 10.1021/nn500897c. 24824726

[ref13] Talapin D. V.; Lee J. S.; Kovalenko M. V.; Shevchenko E. V. Prospects of Colloidal Nanocrystals for Electronic and Optoelectronic Applications. Chem. Rev. 2010, 110, 389–458. 10.1021/cr900137k. 19958036

[ref14] Talapin D. V.; Murray C. B. Applied Physics: PbSe Nanocrystal Solids for n- and p-Channel Thin Film Field-Effect Transistors. Science 2005, 310, 86–89. 10.1126/science.1116703. 16210533

[ref15] Kovalenko M. V.; Bodnarchuk M. I.; Zaumseil J.; Lee J. S.; Talapin D. V. Expanding the Chemical Versatility of Colloidal Nanocrystals Capped with Molecular Metal Chalcogenide Ligands. J. Am. Chem. Soc. 2010, 132, 10085–10092. 10.1021/ja1024832. 20593874

[ref16] Giansante C.; Infante I.; Fabiano E.; Grisorio R.; Suranna G. P.; Gigli G. Darker-than-Black” PbS Quantum Dots: Enhancing Optical Absorption of Colloidal Semiconductor Nanocrystals via Short Conjugated Ligands. J. Am. Chem. Soc. 2015, 137, 1875–1886. 10.1021/ja510739q. 25574692

[ref17] Krieg F.; Ochsenbein S. T.; Yakunin S.; Ten Brinck S.; Aellen P.; Süess A.; Clerc B.; Guggisberg D.; Nazarenko O.; Shynkarenko Y.; Kumar S.; Shih C. J.; Infante I.; Kovalenko M. V. Colloidal CsPbX_3_ (X = Cl, Br, I) Nanocrystals 2.0: Zwitterionic Capping Ligands for Improved Durability and Stability. ACS Energy Lett. 2018, 3, 641–646. 10.1021/acsenergylett.8b00035. 29552638 PMC5848145

[ref18] Zhang B.; Wang M.; Ghini M.; Melcherts A. E. M.; Zito J.; Goldoni L.; Infante I.; Guizzardi M.; Scotognella F.; Kriegel I.; De Trizio L.; Manna L. Colloidal Bi-Doped Cs_2_Ag_1–x_ Na_x_InCl_6_ Nanocrystals: Undercoordinated Surface Cl Ions Limit Their Light Emission Efficiency. ACS Mater. Lett. 2020, 2, 1442–1449. 10.1021/acsmaterialslett.0c00359. 33644762 PMC7901666

[ref19] Ruddigkeit L.; Van Deursen R.; Blum L. C.; Reymond J. L. Enumeration of 166 Billion Organic Small Molecules in the Chemical Universe Database GDB-17. J. Chem. Inf. Model. 2012, 52, 2864–2875. 10.1021/ci300415d. 23088335

[ref20] CAS REGISTRY - The gold standard for chemical substance information. https://www.cas.org/support/documentation/chemical-substances.

[ref21] van Beek B. nlesc-nano/auto-FOX: Auto-FOX 0.8.7; Zenodo, 10.5281/zenodo.4238447.

[ref22] Jones J. E. On the Determination of Molecular Fields.—I. From the Variation of the Viscosity of a Gas with Temperature. Proc. R. Soc. London, Ser. A 1924, 106, 441–462. 10.1098/rspa.1924.0081.

[ref23] Jones J. E. On the Determination of Molecular Fields.—II. From the Equation of State of a Gas. Proc. R. Soc. London, Ser. A 1924, 106, 463–477. 10.1098/rspa.1924.0082.

[ref24] Parr R. G.; Yang W. Density-Functional Theory of Atoms and Molecules; Oxford University Press, 1989.

[ref25] Case D. A.; Cheatham T. E.; Darden T.; Gohlke H.; Luo R.; Merz K. M.; Onufriev A.; Simmerling C.; Wang B.; Woods R. J. The Amber Biomolecular Simulation Programs. J. Comput. Chem. 2005, 26, 1668–1688. 10.1002/jcc.20290. 16200636 PMC1989667

[ref26] Brooks B. R.; Brooks C. L. III; Mackerell A. D.; Nilsson L.; Petrella R. J.; Roux B.; Won Y.; Archontis G.; Bartels C.; Boresch S.; Caflisch A.; Caves L.; Cui Q.; Dinner A. R.; Feig M.; Fischer S.; Gao J.; Hodoscek M.; Im W.; Kuczera K.; Lazaridis T.; Ma J.; Ovchinnikov V.; Paci E.; Pastor R. W.; Post C. B.; Pu J. Z.; Schaefer M.; Tidor B.; Venable R. M.; Woodcock H. L.; Wu X.; Yang W.; York D. M.; Karplus M. CHARMM: The Biomolecular Simulation Program. J. Comput. Chem. 2009, 30, 1545–1614. 10.1002/jcc.21287. 19444816 PMC2810661

[ref27] Berendsen H. J. C.; van der Spoel D.; van Drunen R. GROMACS: A Message-Passing Parallel Molecular Dynamics Implementation. Comput. Phys. Commun. 1995, 91, 43–56. 10.1016/0010-4655(95)00042-E.

[ref28] Yang Y.; Qin H.; Peng X. Intramolecular Entropy and Size-Dependent Solution Properties of Nanocrystal–Ligands Complexes. Nano Lett. 2016, 16, 2127–2132. 10.1021/acs.nanolett.6b00737. 26923516

[ref29] Yang Y.; Qin H.; Jiang M.; Lin L.; Fu T.; Dai X.; Zhang Z.; Niu Y.; Cao H.; Jin Y.; Zhao F.; Peng X. Entropic Ligands for Nanocrystals: From Unexpected Solution Properties to Outstanding Processability. Nano Lett. 2016, 16, 2133–2138. 10.1021/acs.nanolett.6b00730. 26923682

[ref30] Bickelhaupt F. M.; Baerends E. J. Kohn–Sham Density Functional Theory: Predicting and Understanding Chemistry. Rev. Comput. Chem. 2007, 15, 1–86. 10.1002/9780470125922.ch1.

[ref31] Pang Z.; Zhang J.; Cao W.; Kong X.; Peng X. Partitioning Surface Ligands on Nanocrystals for Maximal Solubility. Nat. Commun. 2019, 10, 2454 10.1038/s41467-019-10389-5. 31165734 PMC6549164

[ref32] Quarta D.; Imran M.; Capodilupo A. L.; Petralanda U.; Van Beek B.; De Angelis F.; Manna L.; Infante I.; De Trizio L.; Giansante C. Stable Ligand Coordination at the Surface of Colloidal CsPbBr_3_ Nanocrystals. J. Phys. Chem. Lett. 2019, 10, 3715–3726. 10.1021/acs.jpclett.9b01634. 31244273

